# Magnetic resonance spectroscopy assessment of brain injury after moderate hypothermia in neonatal encephalopathy: a prospective multicentre cohort study

**DOI:** 10.1016/S1474-4422(18)30325-9

**Published:** 2019-01

**Authors:** Peter J Lally, Paolo Montaldo, Vânia Oliveira, Aung Soe, Ravi Swamy, Paul Bassett, Josephine Mendoza, Gaurav Atreja, Ujwal Kariholu, Santosh Pattnayak, Palaniappan Sashikumar, Helen Harizaj, Martin Mitchell, Vijayakumar Ganesh, Sundeep Harigopal, Jennifer Dixon, Philip English, Paul Clarke, Priya Muthukumar, Prakash Satodia, Sarah Wayte, Laurence J Abernethy, Kiran Yajamanyam, Alan Bainbridge, David Price, Angela Huertas, David J Sharp, Vaneet Kalra, Sanjay Chawla, Seetha Shankaran, Sudhin Thayyil, MARBLE consortium, Peter J Lally, Paolo Montaldo, Vânia Oliveira, Aung Soe, Ravi Swamy, Paul Bassett, Josephine Mendoza, Gaurav Atreja, Ujwal Kariholu, Santosh Pattnayak, Palaniappan Sashikumar, Helen Harizaj, Martin Mitchell, Vijayakumar Ganesh, Sundeeep Harigopal, Jennifer Dixon, Philip English, Paul Clarke, Priya Muthukumar, Prakash Satodia, Sarah Wayte, Laurence J Abernethy, Kiran Yajamanyam, Alan Bainbridge, David Price, Angela Huertas, David J Sharp, Vaneet Kalra, Sanjay Chawla, Seetha Shankaran, Sudhin Thayyil

**Affiliations:** a Centre for Perinatal Neuroscience, Imperial College London, London, UK; b Oliver Fisher Neonatal Unit, Medway NHS Foundation Trust, Kent, UK; c Statsconsultancy, Amersham, UK; d Neonatal Unit, Imperial College Healthcare NHS Trust, London, UK; e Neonatal Unit, Royal Victoria Infirmary, Newcastle, UK; f Neonatal Unit, Norfolk and Norwich University Hospitals NHS Foundation Trust, Norwich, UK; g Neonatal Unit, University Hospitals Coventry and Warwickshire NHS Trust, Coventry, UK; h Neonatal Unit, Liverpool Women's NHS Foundation Trust, Liverpool, UK; i Neonatal Unit, University College London Hospitals NHS Foundation Trust, London, UK; j Computational, Cognitive and Clinical Neuroimaging Laboratory, Imperial College London, London, UK; k Neonatal-Perinatal Medicine, Wayne State University, Detroit, MI, USA

## Abstract

**Background:**

In neonatal encephalopathy, the clinical manifestations of injury can only be reliably assessed several years after an intervention, complicating early prognostication and rendering trials of promising neuroprotectants slow and expensive. We aimed to determine the accuracy of thalamic proton magnetic resonance (MR) spectroscopy (MRS) biomarkers as early predictors of the neurodevelopmental abnormalities observed years after neonatal encephalopathy.

**Methods:**

We did a prospective multicentre cohort study across eight neonatal intensive care units in the UK and USA, recruiting term and near-term neonates who received therapeutic hypothermia for neonatal encephalopathy. We excluded infants with life-threatening congenital malformations, syndromic disorders, neurometabolic diseases, or any alternative diagnoses for encephalopathy that were apparent within 6 h of birth. We obtained T_1_-weighted, T_2_-weighted, and diffusion-weighted MRI and thalamic proton MRS 4–14 days after birth. Clinical neurodevelopmental tests were done 18–24 months later. The primary outcome was the association between MR biomarkers and an adverse neurodevelopmental outcome, defined as death or moderate or severe disability, measured using a multivariable prognostic model. We used receiver operating characteristic (ROC) curves to examine the prognostic accuracy of the individual biomarkers. This trial is registered with ClinicalTrials.gov, number NCT01309711.

**Findings:**

Between Jan 29, 2013, and June 25, 2016, we recruited 223 infants who all underwent MRI and MRS at a median age of 7 days (IQR 5–10), with 190 (85%) followed up for neurological examination at a median age of 23 months (20–25). Of those followed up, 31 (16%) had moderate or severe disability, including one death. Multiple logistic regression analysis could not be done because thalamic N-acetylaspartate (NAA) concentration alone accurately predicted an adverse neurodevelopmental outcome (area under the curve [AUC] of 0·99 [95% CI 0·94–1·00]; sensitivity 100% [74–100]; specificity 97% [90–100]; n=82); the models would not converge when any additional variable was examined. The AUC (95% CI) of clinical examination at 6 h (n=190) and at discharge (n=167) were 0·72 (0·65–0·78) and 0·60 (0·53–0·68), respectively, and the AUC of abnormal amplitude integrated EEG at 6 h (n=169) was 0·73 (0·65–0·79). On conventional MRI (n=190), cortical injury had an AUC of 0·67 (0·60–0·73), basal ganglia or thalamic injury had an AUC of 0·81 (0·75–0·87), and abnormal signal in the posterior limb of internal capsule (PLIC) had an AUC of 0·82 (0·76–0·87). Fractional anisotropy of PLIC (n=65) had an AUC of 0·82 (0·76–0·87). MRS metabolite peak-area ratios (n=160) of NAA–creatine (<1·29) had an AUC of 0·79 (0·72–0·85), of NAA–choline had an AUC of 0·74 (0·66–0·80), and of lactate–NAA (>0·22) had an AUC of 0·94 (0·89–0·97).

**Interpretation:**

Thalamic proton MRS measures acquired soon after birth in neonatal encephalopathy had the highest accuracy to predict neurdevelopment 2 years later. These methods could be applied to increase the power of neuroprotection trials while reducing their duration.

**Funding:**

National Institute for Health Research UK.

## Introduction

Neonatal encephalopathy affects 2–8 in every 1000 livebirths and carries a high risk of death or severe disability.^1^ Therapeutic hypothermia has been shown to reduce this risk but many treated infants still go on to develop substantial lifelong disabilities.^2^

In neuroprotective trials that target neonatal encephalopathy, there is a crucial difficulty in the assessment of treatment efficacy: an accurate clinical measurement of neurodevelopment can only be taken after a delay of several years. Currently, more than a dozen putative neuroprotectants are awaiting clinical evaluation on the evidence of promising preclinical work.^3^ Thalamic proton magnetic resonance (MR) spectroscopy (MRS), obtained within days or weeks of the initial injury, gives insight into the metabolic effects of neonatal encephalopathy and hence could be useful in quantifying treatment effects of neuroprotective therapies.^4^, ^5^, ^6^

**Research in context**

**Evidence before this study**

We searched MEDLINE (PubMed interface) and Embase without any language restrictions for articles published from Jan 1, 1990, to May 1, 2018, using Medical Subject Heading terms and the following keywords: [magnetic resonance imaging OR magnetic resonance spectroscopy] AND [hypoxic ischemic encephalopathy] AND [newborn OR neurodevelopmental outcome]. We also examined the references and bibliographies from retrieved articles. 42 single-centre prospective or retrospective studies (the largest prospective study including 33 infants) compared proton magnetic resonance (MR) spectroscopy (MRS) metabolite peak-area ratios in infants with neonatal encephalopathy with neurodevelopmental outcome at 12 months or older, of which four also examined absolute metabolite concentrations. Four retrospective studies compared the proton MRS in encephalopathic infants who had therapeutic hypothermia with later neurodevelopmental outcomes, of which only two used 3·0 Tesla MR scanners. Although all the studies reported that proton MRS was a good predictor of neurodevelopmental outcome, the MR acquisition details, voxel positioning, scanner types, and prognostic cutoff thresholds varied widely. The lack of generalisability in these studies therefore resulted in minimal clinical impact.

We found no published data of proton MRS studies using different MR scanner models across different vendors, with harmonised acquisitions. Three recent systematic reviews of the published literature on MRS in neonatal encephalopathy, and a consensus group of MRS experts, concluded that the absence of such multicentre proton MRS studies is the biggest roadblock preventing the mainstream clinical application of MRS.

**Added value of this study**

To our knowledge, this is the first large prospective and pragmatic multicentre study assessing the prognostic accuracy of proton MRS in neonatal encephalopathy. This investigation involved clinical 3·0 Tesla MR systems from three prominent vendors, to ensure widespread applicability to pragmatic clinical settings. In this context, we showed that the thalamic concentration of N-acetylaspartate (NAA), measured within 14 days after birth, accurately predicted adverse neurodevelopmental outcomes 2 years later. Proton MRS biomarkers, independent of any other measures, gave a substantial improvement in prognostic accuracy over available clinical measures and conventional MRI scoring.

**Implications of all the available evidence**

Proton MRS should be a routine component of clinical MR protocols for infants with neonatal encephalopathy. This assessment is ideal for early prognostication and might provide promising surrogate outcome measures for multicentre clinical trials. If early-phase trials were to employ proton MRS measures such as NAA concentration, this would greatly increase their power to detect important treatment effects while potentially reducing their duration by several years.

Although meta-analyses have shown that proton MRS provides accurate prognosis in neonatal encephalopathy across various single-centre studies,^7^, ^8^ it is challenging to achieve similar results across a network of institutions with different MR scanners.^9^ No such studies have been done in pragmatic multicentre clinical settings, as is required for large neuroprotection trials. Because each MR scanner is supplied with its own proprietary software for clinical use, each having its own hardware and software configuration, substantial work is required to ensure any quantitative results are comparable across institutions. Thus, despite the promise and wide availability of proton MRS on all modern scanners, it has not yet entered mainstream use in clinical practice.

The expert MRS Consensus Group has highlighted several key pathologies where proton MRS can have a substantial impact on the clinical management of patients, including neonatal encephalopathy, but concludes that important barriers need to be overcome to achieve this routinely.^10^ Particularly, they highlight the need for prospective multicentre clinical trials with robust standardisation, analysis, and quality assurance. In this study, our aim was to harmonise the acquisition of robust thalamic proton MRS biomarkers across several tertiary neonatal care centres and assess their accuracy in predicting adverse neurodevelopment in term or near-term infants 2 years after neonatal encephalopathy.

## Methods

### Study design and participants

The magnetic resonance biomarkers in neonatal encephalopathy (MARBLE) study recruited consecutive term and near-term infants (36–43 weeks' gestation) with neonatal encephalopathy who received therapeutic hypothermia, with staggered start dates across eight participating neonatal intensive care units in the UK and USA. Infants were excluded if they had life-threatening congenital malformations, syndromic disorders, or neurometabolic diseases. The study was approved by the North London Research Ethics Committee (13HH1843), and each of the participating clinical sites. All parents provided written informed consent, as stipulated by the ethics committee.^11^ The study protocol is available online.

### Procedures

Upon recruitment at each site, infants were assessed according to the National Institute of Child Health and Human Development (NICHD) neurological examination with an additional categorisation for mild encephalopathy.^12^ This yielded an encephalopathy grade (none, mild, moderate, or severe) which reflected the severity of neurological symptoms across six categories (appendix).

For the study, we did MR brain scans 4–14 days after birth on a 3.0 Tesla scanner (Philips [Amsterdam, Netherlands], Siemens [Munich, Germany], or GE Healthcare [Chicago, IL, USA]), with harmonised protocols.^11^ The MR protocol (acquisition time) comprised T_1_-weighted and T_2_-weighted MRI (15 min) and diffusion-weighted MRI (DW-MRI; 7 min), proton MRS metabolite peak-area ratios (7 min), and proton MRS metabolite absolute concentrations (25 min). We did MRS in a single 15 × 15 × 15 mm^3^ voxel centred on the left thalamus (appendix). Pseudonymised MR data were transferred to Imperial College London (London, UK) for post-processing and analysis. An MR physicist (PJL) analysed the MRS and DW-MRI data, and a neonatal neurologist (ST) with 10 years of MR experience reported the conventional MRI data using a validated scoring system^13^ while masked to the clinical outcomes (appendix).

We classified the amplitude integrated EEG (aEEG) background activity as normal (upper margin of aEEG activity band >10 μV and lower margin >5 μV); moderately abnormal amplitude (upper margin >10 μV and lower margin <5 μV); or severely abnormal amplitude (upper margin <10 μV and lower margin <5 μV).^14^

We assessed neurodevelopmental outcome using the Bayley Scales of Infant and Toddler Development, third edition (Bayley-III) cognitive, language, and motor composite scores and a detailed neurological examination by trained and certified examiners who were masked to the MRS and DW-MRI data. We defined severe disability as any of the following: Bayley-III composite cognitive and language scores less than 70, Gross Motor Function Classification System (GMFCS) levels 3–5, hearing impairment requiring hearing aids, or blindness. We defined moderate disability as composite cognitive and language scores between 70 and 84, and any of the following: GMFCS level 2, hearing impairment without the need for amplification, or a persistent seizure disorder.

### Outcomes

The primary objective was to examine the accuracy of quantitative cerebral MR biomarkers for predicting adverse 18–24-month neurodevelopmental outcome, as measured by a composite of death or moderate or severe disability in survivors after therapeutic hypothermia for neonatal encephalopathy.

Secondary outcomes (which are not reported here) were intercentre variability of proton MRS measurements and the incremental benefits of quantitative MR biomarkers for predicting adverse outcomes when compared with conventional MRI assessment and bedside assessments and investigations.

We selected the following key MR biomarkers of interest: thalamic concentration of N-acetylaspartate (NAA); peak-area ratios of thalamic lactate to NAA, NAA to creatine, and NAA to choline; basal ganglia or thalamic injury or cortical injury on T_1_-weighted and T_2_-weighted MRI; loss of the normal high signal intensity of the posterior limb of internal capsule (PLIC) on T_1_-weighted MRI; and fractional anisotropy in the PLIC (from DW-MRI); in addition to a structured clinical neurological examination and moderate or severe voltage abnormality of aEEG within the first 6 h of age and at discharge. We considered all these markers for inclusion in a single multivariable prognostic model in predicting an adverse outcome at 2 years.

### Statistical analysis

We calculated the sample size using the so-called rule of ten generally used to calculate the sample size requirements for developing prognostic models. The multivariable risk model required a total sample size of 180, assuming an adverse outcome in 50% of participants. We increased the sample size to 220 to account for attrition and poor data quality. Comparisons of continuous biomarkers between infants with normal and adverse outcomes were done using the unpaired *t* test if the variables were normally distributed or the Mann-Whitney test if they were not.

We also used receiver operating characteristic (ROC) curves to examine the prognostic accuracy of these biomarkers and obtained prognostic indices with 95% CIs using the exact binomial method.

Finally, we used linear regression to examine the association between the nine key biomarkers and the cognitive, language, and motor composite scores obtained with Bayley-III. Where infants had severe disability, irrespective of encephalopathy grade, that prevented them from undertaking Bayley-III, we assigned nominal values for the composite scores in each domain (cognitive=54, language=46, motor=46). We examined the association between each biomarker and Bayley-III scores in separate linear regression analyses, followed by a multivariable analysis to examine the joint association. We employed a backwards selection procedure to retain only statistically significant factors in the final models. Due to its skewed distribution, we analysed the peak-area ratio of lactate to NAA on the log scale. We adjusted all regression analyses for birth gestational age and postnatal age at MRI.

This trial is registered with ClinicalTrials.gov, number NCT01309711.

### Role of the funding source

The funders of the study had no role in study design, data collection, data analysis, data interpretation, or writing of the report. The corresponding author had full access to all the data in the study and the final decision to submit for publication. All authors approved the final version of the manuscript submitted for publication.

## Results

**Figure 1:**
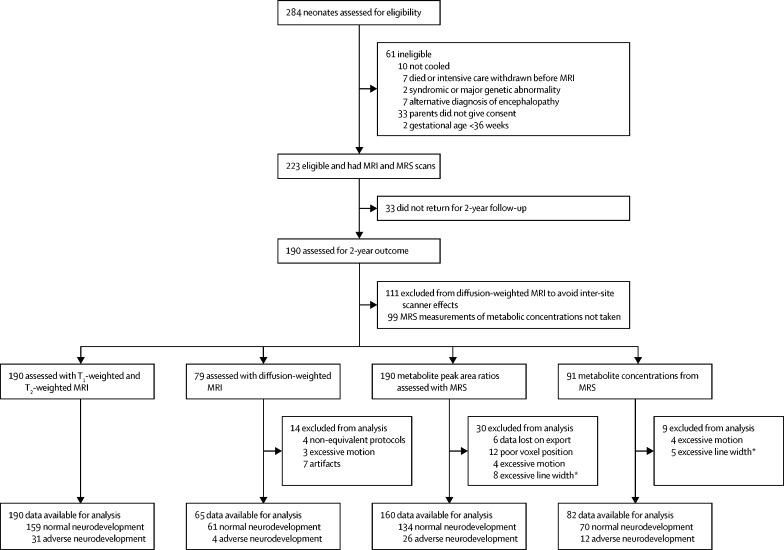
Study profile MRS=magnetic resonance spectroscopy. *Excessive line width defined as greater than mean + 2SD.

Between Jan 29, 2013, and June 25, 2016, MARBLE recruited a total of 223 infants, of whom 190 (85%) had neurodevelopmental outcome assessments for analysis at a median age of 23 months (IQR 20–25). Of those infants, 190 (100%) had conventional MRI, 160 (84%) had proton MRS including thalamic metabolite peak-area ratios (ie, lactate–NAA and NAA–creatine ratios), 82 (43%) had proton MRS including thalamic NAA concentration, and 65 (34%) had DW-MRI data for analysis, following quality control (figure 1). Of the infants with outcome data, 31 (16%) had an adverse neurodevelopmental outcome (death or moderate or severe disability). The clinical characteristics, brain injury, and neurodevelopmental outcomes of infants who had thalamic NAA concentration measurements were similar to those who did not (appendix).

Across all eligible infants, the mean birthweight was 3·4 kg (SD 0·6), at a mean gestational age of 39·9 weeks (1·5), with 216 infants undergoing 72 h of moderate hypothermia (33·5°C), six infants being rewarmed prior to 72 h, and one infant being rewarmed after 96 h due to local clinical decisions (table 1). According to assessment against NICHD criteria within the first 6 h after birth, encephalopathy was graded severe in 23 (10%) infants, moderate in 163 (73%), and mild in 36 (16%). In one more case, the infant was initially assessed as having no encephalopathy at admission but this progressed within the first 6 h to mild neonatal encephalopathy and a clinical decision was made to begin hypothermia (table 2). MR scanning was done at a mean age of 8·4 days (SD 4·3), which did not differ between infants who went on to have normal or adverse outcomes (p>0·5; Mann-Whitney *U*).

**Table 1:** Baseline clinical characteristics

			**Total participants (n=223)**	**Normal outcome (n=159)**	**Adverse outcome (n=31)**	**Lost to follow-up (n=33)**
Primigravida			104/219 (47%)	79/156 (51%)	13/31 (42%)	12/32 (38%)
Reduced fetal movements			30/175 (17%)	19/123 (15%)	9/26 (35%)	2/26 (8%)
Prolonged rupture of membranes			36/214 (17%)	25/152 (16%)	6/31 (19%)	5/31 (16%)
Abnormal cardiotocogram			112/137 (82%)	84/105 (80%)	11/13 (85%)	17/19 (89%)
Antepartum haemorrhage			15/213 (7%)	9/153 (6%)	4/29 (14%)	2/31 (6%)
Meconium stained liquor			76/214 (36%)	59/152 (39%)	8/30 (27%)	9/32 (28%)
Emergency LSCS			92/217 (42%)	65/154 (42%)	14/31 (45%)	13/32 (41%)
Delivery complications						
	Uterine rupture		11/215 (5%)	6/155 (4%)	4 (13%)	1/29 (3%)
	Cord prolapse		7/215 (3%)	4/155 (3%)	2 (6%)	1/29 (3%)
	Shoulder dystocia		26/215 (12%)	14/155 (9%)	6 (19%)	6/29 (21%)
	Obstructed labour		4/215 (2%)	3/155 (2%)	1 (3%)	0
Birth and resuscitation						
	Sex					
		Female	99 (44%)	69 (43%)	14 (45%)	16 (48%)
		Male	124 (56%)	90 (57%)	17 (55%)	17 (52%)
	Birthweight, kg*		3·4 (0·6)	3·4 (0·5)	3·2 (0·6)	3·5 (0·5)
	Gestation, weeks*		39·9 (1·5)	39·9 (1·5)	39·6 (1·4)	40·0 (1·6)
	Cord pH*		6·9 (1·9)	7·0 (1·8)	6·9 (2·4)	6·9 (1·8)
	Apgar score, 10 min*		5·6 (2·2)	5·7 (2·2)	5·0 (2·3)	5·4 (2·3)
	Intubation		68/217 (31%)	49/155 (32%)	8/30 (27%)	11/32 (34%)
	Cardiac massage		53/217 (24%)	39/155 (25%)	10/30 (33%)	4/32 (13%)
	Drugs (adrenaline, bicarbonate)		9/217 (4%)	5/155 (3%)	2/30 (7%)	2/32 (6%)
Admission encephalopathy stage						
	None		1 (<1%)†	1 (1%)†	0	0
	Mild		36 (16%)	29 (18%)	1 (3%)	6 (18%)
	Moderate		163 (73%)	119 (75%)	17 (55%)	27 (82%)
	Severe		23 (10%)	10 (6%)	13 (42%)	0
Neonatal course						
	Coagulopathy		57/208 (27%)	35/148 (24%)	10/28 (36%)	12/32 (38%)
	Positive blood culture		12/204 (6%)	8/144 (6%)	3/28 (11%)	1 (3%)
	Pneumothorax		12/215 (6%)	10/152 (7%)	1/30 (3%)	1 (3%)
	Hypotension		88/218 (40%)	53/154 (34%)	19 (61%)	16 (48%)
	Persistent pulmonary hypertension		18/216 (8%)	14/153 (9%)	0	4 (12%)
	Pulmonary haemorrhage		3/215 (1%)	2/152 (1%)	0	1 (3%)
	Seizures on admission		115/210 (55%)	78/149 (52%)	21/30 (70%)	16/31 (52%)
	Age at MRI scan, days*		8·4 (4·3)	8·4 (4·6)	8·8 (3·4)	7·8 (3·3)

Data are n (%), n/N (%), or mean (SD). Percentages are in terms of the number of mothers or neonates for whom data were available. LSCS=lower segment caesarean section.

* Birthweight measured in 216 total participants (154 normal outcome, 29 adverse outcome, 33 lost to follow-up). Gestation measured in 218 total participants (155 normal outcome, 30 adverse outcome, 33 lost to follow-up). Cord pH measured in 194 total participants (140 normal outcome, 29 adverse outcome, 25 lost to follow-up). Apgar score measured in 178 total participants (130 normal outcome, 26 adverse outcome, 22 lost to follow-up. Age at MRI scan available for all participants.

† Progressed to moderate encephalopathy and hence cooled.

**Table 2:** Brain injury and neurodevelopmental outcome according to the initial severity of encephalopathy

		Mild neonatal encephalopathy (n=37)*	**Moderate neonatal encephalopathy (n=163)**	**Severe neonatal encephalopathy (n=23)**
Basal ganglia or thalamic injury score				
	0	36 (97%)	128 (79%)	14 (61%)
	1	1 (3%)	16 (10%)	3 (13%)
	2	0	12 (7%)	5 (22%)
	3	0	7 (4%)	1 (4%)
White matter score				
	0	17 (46%)	51 (31%)	9 (39%)
	1	16 (43%)	64 (39%)	4 (17%)
	2	4 (11%)	36 (22%)	8 (35%)
	3	0	12 (7%)	2 (9%)
Cortical score				
	0	33 (89%)	126 (77%)	15 (65%)
	1	4 (11%)	23 (14%)	6 (26%)
	2	0	6 (4%)	1 (4%)
	3	0	8 (5%)	1 (4%)
PLIC score				
	Normal	37 (100%)	133 (82%)	12 (52%)
	Equivocal	0	18 (11%)	4 (17%)
	Abnormal	0	12 (7%)	7 (30%)
NAA concentration, mmol/kg wet weight†		7·3 (0·9)	6·8 (1·3)	5·1 (1·4)
Median lactate–NAA ratio (IQR)†		0·12 (0·10–0·14)	0·15 (0·11–0·20)	0·25 (0·18–0·65)
NAA–creatine ratio†		1·62 (0·19)	1·53 (0·25)	1·37 (0·38)
NAA–choline ratio†		0·87 (0·13)	0·83 (0·16)	0·78 (0·16)
Outcome data between 18 to 24 months available		31 (84%)	136 (83%)‡	23 (100%)
Age at the outcome assessment, months†		23·5 (4·2)	23·5 (3·9)	22·5 (2·4)
Adverse outcome		1/31 (3%)§	17 (10%)	13 (57%)
Median GMFCS (IQR)†		0·0 (0·0–0·0)	0·0 (0·0–0·0)	3·0 (0·0–4·0)
Bayley-III composite cognitive score†		105·1 (15·3)	98·5 (18·3)	73·6 (22·5)
Bayley-III composite language score†		98·0 (14·4)	92·4 (18·6)	65·1 (23·0)
Bayley-III composite motor score†		100·3 (9·8)	95·6 (19·3)	68·7 (26·5)
Cerebral palsy		1 (3%)	19 (12%)	12 (52%)
Visual problems		0/27	6/120 (5%)	3/22 (14%)
Hearing problems		0/27	3/120 (3%)	3/22 (14%)
Epilepsy		0/27	8/122 (7%)	2/22 (9%)

All data are n (%), n/N (%), or mean (SD) unless otherwise specified. Percentages are based on the number of infants for whom the data were available. Basal ganglia or thalamic injury, white matter, and cortical scores are defined as follows: 0=normal, 1=mild injury, 2=moderate injury, 3=severe injury. Further details of the scoring system are given in the appendix. Bayley-III=Bayley Scales of Infant and Toddler Development, third edition. GMFCS=gross motor function classification system. NAA=N-acetylaspartate. PLIC=posterior limb of internal capsule.

* One baby had no encephalopathy at 6 h but developed seizures at 10 h and was cooled.

† NAA concentration measured in 16 infants with mild, 67 with moderate, and nine with severe encephalopathy. Peak-area ratios measured in 31 infants with mild, 136 infants with moderate, and 22 infants with severe encephalopathy. Age at the outcome assessment at 18–24 months available for 37 infants with mild, 163 infants with moderate, and 23 infants with severe encephalopathy. GMFCS assessed in 30 infants with mild, 136 with moderate, and 23 with severe encephalopathy. Bayley-III cognitive score measured in 28 infants with mild, 126 with moderate, and 21 with severe encephalopathy. Bayley-III language score measured in 27 infants with mild, 121 with moderate, and 20 with severe encephalopathy. Bayley-III motor score measured in 28 infants with mild, 126 with moderate, and 21 with severe encephalopathy. Visual and hearing problems measured in 27 infants with mild, 120 with moderate, and 22 with severe encephalopathy. Data on epilepsy available in 25 infants with mild, 122 with moderate, and 22 with severe encephalopathy.

‡ One infant died at 20 months.

§ Rewarmed before completing 72 h of cooling due to rapid clinical improvement.

**Figure 2:**
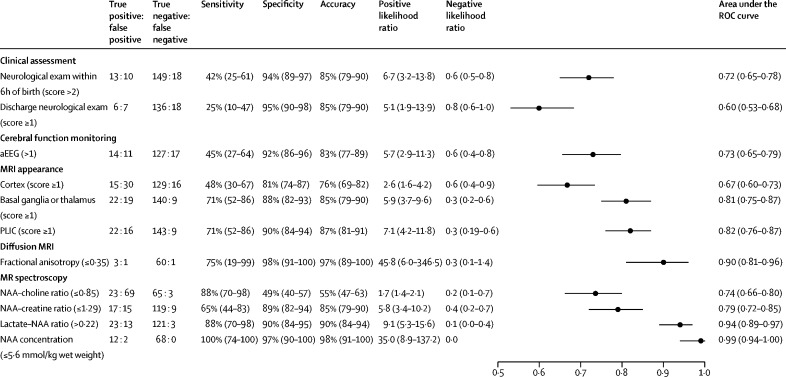
Prognostic accuracy of biomarkers for predicting adverse neurodevelopmental outcomes at 2 years Parentheses and error bars indicate 95% CIs. Scores are defined as follows: discharge neurological exam: 1=mild encephalopathy, 2=moderate encephalopathy, 3=severe encephalopathy; aEEG: 0=normal amplitude, 1=moderately abnormal amplitude, 2=severely abnormal amplitude; MRI appearance of cortex or basal basal ganglia or thalamus: 0=normal, 1=mild injury, 2=moderate injury, 3=severe injury; and PLIC: 0=normal, 1=equivocal, 2=abnormal. aEEG=amplitude integrated EEG. NAA=N-acetylaspartate. PLIC=posterior limb of internal capsule. ROC=receiver operating characteristic.

The prognostic accuracy of the biomarkers in predicting an adverse outcome is shown in figure 2, with ROC curves of the most commonly used biomarkers shown in the appendix. Clinical neurological examination at discharge had a good specificity but poor sensitivity (figure 2). This had poorer prognostic accuracy than the equivalent neurological examination done within 6 h of birth, with a sensitivity of 42% (95% CI 25–61) and specificity of 94% (89–97). aEEG done within the first 6 h of birth had a similar sensitivity and specificity to the neurological examination done within 6 h of birth (figure 2).

From T_1_-weighted and T_2_-weighted MRI, the most predictive measure was any evidence of injury to either the PLIC or the basal ganglia or thalamus (figure 2).

**Figure 3:**
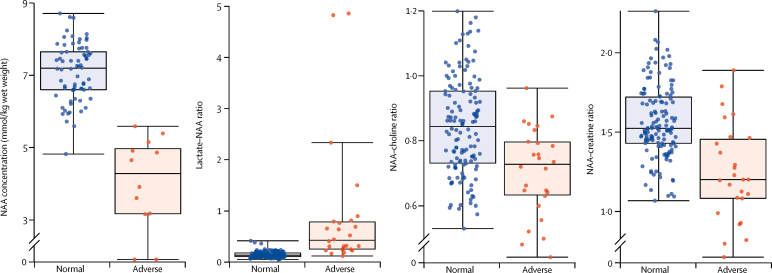
Box plots of proton MRS biomarker values for children with normal compared with adverse neurodevelopmental outcome at 2 years Box plots show the spread of the datapoints overlaying the median and IQR. Medians are indicated by horizontal lines; boxes outline the upper and lower quartiles; and the whiskers indicate 1·5×IQR from upper and lower quartiles. Outliers are indicated with dots lying beyond the whiskers. p<0·0001 for all analyses. NAA=N-acetylaspartate.

Thalamic NAA concentration was the single best prognostic indicator, with an area under the curve (AUC) of 0·99 (95% CI 0·94–1·00) and high sensitivity and specificity (figure 2). The mean NAA concentration in infants with normal outcomes was 7·1 mmol/kg wet weight (SD 0·8) and 4·0 mmol/kg wet weight (1·3) in those with adverse outcomes (p<0·0001; *t* test). Although NAA concentration increased with gestational age at birth (Pearson correlation coefficient *r* = 0·31; p=0·0031), there was no relation with postnatal age (*r* = 0·02; p=0·83). Neither gestational age nor postnatal age at the MR scan were associated with adverse outcomes (appendix). The lactate–NAA peak-area ratio had an AUC of 0·94 (0·89–0·97; figure 2). The median lactate–NAA peak-area ratio was 0·14 (IQR 0·11–0·18) in infants with a normal outcome and 0·43 (0·25–0·79) in infants with an adverse outcome (p<0·0001; Mann-Whitney *U*). Mean NAA–creatine peak-area ratios in infants with normal and adverse outcomes was 1·56 (SD 0·23) in infants with normal outcomes and 1·25 (0·31) in those with adverse outcomes whereas mean NAA–choline peak-area ratios were 0·84 (0·15) in infants with normal outcomes and 0·71 (0·13) in those with adverse outcomes (p<0·0001 for both analyses; *t* test). Figure 3 shows the thalamic metabolite distributions and outcomes. NAA concentration was normally distributed and showed a clear separation between infants with normal and adverse outcomes. By contrast, the lactate–NAA ratio showed a highly skewed distribution with clustering around a narrow range.

**Figure 4:**
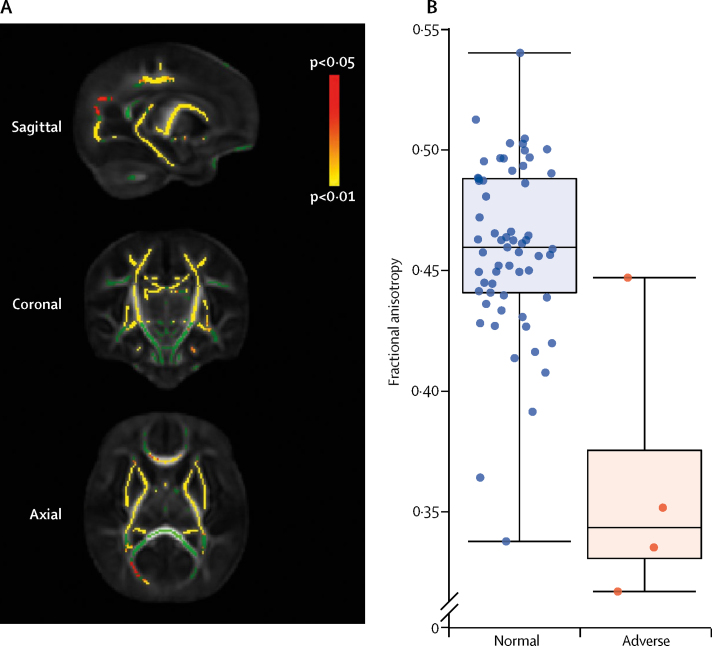
Tract-based spatial statistical analysis results of whole-brain white matter fractional anisotropy (A) and the box plot of the fractional anisotropy values across the posterior limb of internal capsule in the normal and adverse neurodevelopmental outcome groups (B) (A) Green indicates regions where there was no statistically significant (p<0·05) groupwise difference in fractional anisotropy between infants with normal or adverse outcomes. Regions in red–yellow indicate regions of increasing statistical significance (p<0·05 in red, p<0·01 in yellow), controlling for corrected gestational age at the time of the scan. (B) Box plots show the spread of the fractional anisotropy datapoints overlaying the median and IQR. Medians are indicated by horizontal lines; boxes outline the upper and lower quartiles; and the whiskers indicate 1·5×IQR from upper and lower quartiles. Outliers are indicated with dots lying beyond the whiskers. Groupwise difference p=0·0054.

From DW-MRI, the fractional anisotropy in the PLICs had an AUC of 0·92 (95% CI 0·76–1·00). The group median fractional anisotropy in the PLICs of infants with adverse outcomes was significantly lower than for those with normal outcomes (0·34 [IQR 0·33–0·38] *vs* 0·46 [0·44–0·49]; p=0·0054; Mann-Whitney *U*; figure 4). Using a groupwise tract-based spatial statistics analysis, and controlling for gestational age at scan, infants who had an adverse neurodevelopmental outcome had lower fractional anisotropy throughout their cerebral white matter (figure 4).

Thalamic NAA concentration alone accurately predicted an adverse outcome, and so multiple logistic regression analysis could not be done because the models would not converge when any additional variable was examined, suggesting the metabolic peak-area ratios were not independently associated with adverse outcomes. It is unlikely a second variable would have been able to improve diagnostic performance.

**Figure 5:**
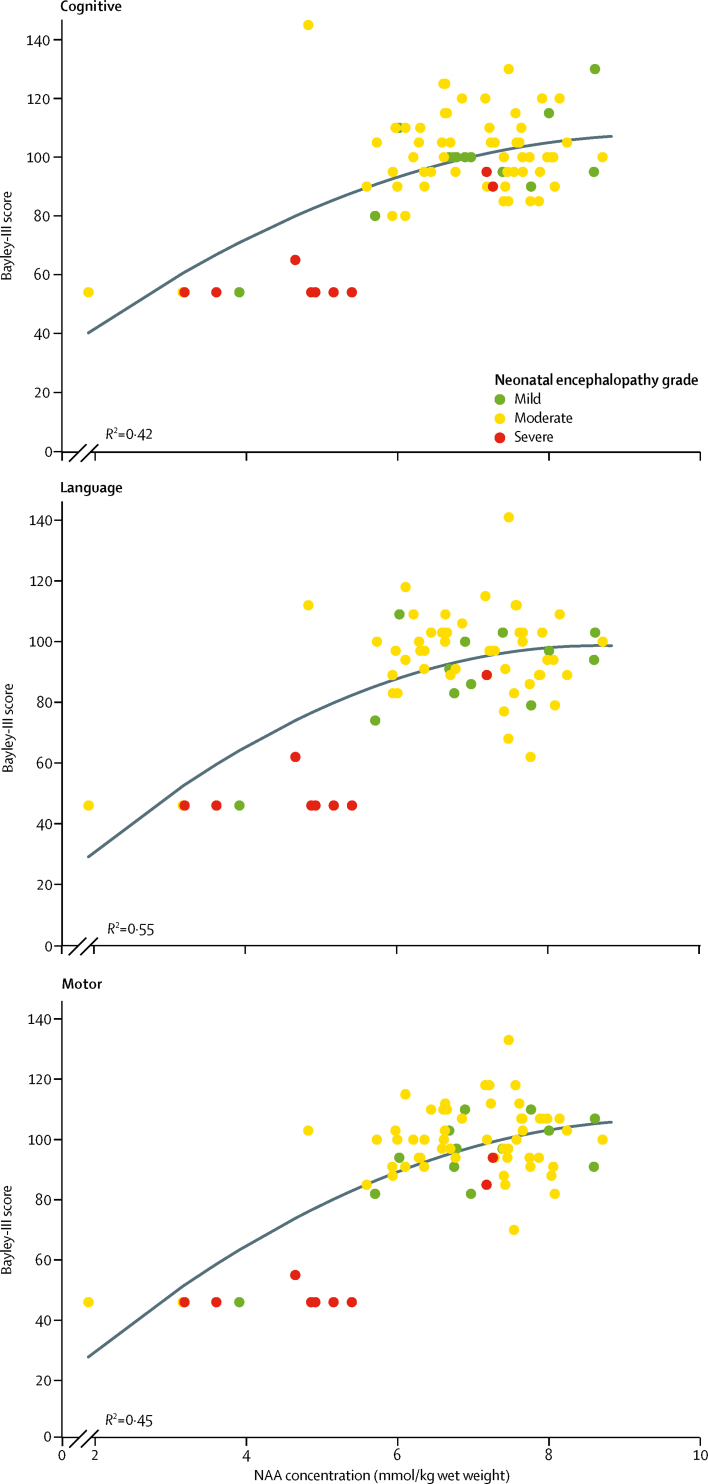
Relation of thalamic NAA concentration with Bayley-III cognitive, motor, and language scores at 2-year follow-up The blue line represents the fitted regression line. Bayley-III=Bayley Scales of Infant and Toddler Development, third edition. NAA=N-acetylaspartate.

Our final set of analyses examined the relationship between each of the MR measures with the continuous neurodevelopmental outcome scores from Bayley-III. When we examined them individually, all nine were significantly associated with these scores (all p<0·0001). Multivariable analyses, summarised in the appendix, suggested that only NAA concentration and PLIC scoring were independently associated with all three outcomes. We found a nonlinear relationship between NAA concentration and each of the scores. We observed a positive association between scores and NAA concentration, although this relation tailed off for high values of NAA concentration (figure 5).

## Discussion

To our knowledge, MARBLE was the largest prospective prognostic study of proton MRS in neonatal encephalopathy, collecting carefully standardised results across multiple sites that reflect the range of MR scanners in clinical use, and employing rigorous quality assurance and centralised analysis. The results provide evidence of the high accuracy of thalamic proton MRS, acquired days after birth, in predicting adverse neurodevelopment 2 years after neonatal encephalopathy. Thalamic NAA concentration had the highest accuracy in predicting neurodevelopmental outcome following neonatal encephalopathy (AUC 0·99, 95% CI 0·94–1·00) and was associated with performance across cognitive, language, and motor domains at 2 years of age. Given this high prognostic accuracy, it was not possible to develop a multivariable prediction model including other parameters.

Although the lactate–NAA peak-area ratio had a sensitivity of 88% (95% CI 70–98) and specificity of 90% (84–95), confirming the results of our previous meta-analysis,^7^ this was overshadowed by the strength of the association between NAA concentration and adverse outcome, which suggests that the lactate–NAA peak-area ratio was not independently associated with adverse outcomes. Thus, the prognostic accuracy of the lactate–NAA peak-area ratio appears to be primarily driven by NAA concentration. Furthermore, unlike NAA concentration, the metabolic information obtained from the lactate–NAA peak-area ratio is ambiguous. Not only is it affected by changes in metabolite concentrations but also their relaxation characteristics and acquisition-dependent chemical shift artifacts. Finally, the diverging time courses of lactate and NAA concentration, the skewed distribution of the lactate–NAA peak-area ratio, and the uncertain effect of cooling therapy on lactate concentration^15^, ^16^, ^17^, ^18^ limits use of the lactate–NAA peak-area ratio as a surrogate endpoint in multicentre trials. However, given the high prognostic accuracy and short acquisition times in routine clinical settings, this ratio would add substantial value to standard clinical imaging protocols. Although NAA concentration increased with gestational age, postnatal age did not influence concentration in infants with neonatal encephalopathy. Hence, the MR scanning could be acquired any time within the first 14 days after birth without affecting the prognostic accuracy.

Clinical examination at birth alone could not accurately identify infants at risk of adverse neurodevelopment, and we found that a second examination at discharge was even less effective. aEEG within 6 h of birth did not enhance the prognostic accuracy of the structured clinical examination, confirming data from a previous meta-analysis^19^ and from cooling trials.^12^ Following hypothermia, conventional MRI is most frequently used in prognostication in clinical settings but its interpretation is subjective and scoring systems are only semiquantitative. In the present study, basal ganglia or thalamic injury on conventional MRI had a sensitivity of 71% (95% CI 52–86) and specificity of 88% (82–93). These results agreed with our previous meta-analysis^7^ of data obtained at a time when moderate hypothermia was still the subject of various randomised controlled trials but had not yet been implemented in widespread clinical practice. Three sub-studies of large cooling trials (TOBY^13^ with 64 participants, NICHD^20^ with 73, and ICE^21^ with 127) have since shown that hypothermia reduces both the frequency and severity of lesions observed on MRI but none of these trials included prognostic measures derived from proton MRS. The prognostic accuracy of conventional MRI was variable and suboptimal in these studies (sensitivities 60–90% and specificities 65–92%) despite a single expert reporting the scans. In routine clinical practice, where scans are often reported by general paediatric or adult neuroradiologists, the accuracy is likely to be even lower.

Of the 190 infants assessed at 2 years after their initial injury, only 31 (16%) had adverse neurodevelopment, which is a lower proportion than we had anticipated when designing the study. This result might reflect improvements attributed to the implementation and rapid initiation of therapeutic hypothermia, and the increased tendency to cool infants with mild encephalopathy.^22^ Many tertiary centres in the UK and other high-income countries now routinely cool infants with mild neonatal encephalopathy.^22^ However, there is little evidence to support such a practice,^23^ and cooling in mild neonatal encephalopathy should not be considered standard care until further animal study and clinical data are available.

Trials examining the optimal duration and delayed initiation of hypothermia in moderate and severe neonatal encephalopathy have also reported lower adverse event rates than expected.^24^, ^25^ The lower frequency of adverse outcomes would require much larger sample sizes for trials based on neurodevelopmental outcome measures, increasing the need for early accurate surrogate endpoints derived from proton MRS. Without exploiting such surrogate endpoints, early phase trials will soon become prohibitively long and expensive. When applied to a population at much higher risk of adverse outcome than in our study, the sensitivity and specificity of thalamic NAA concentration will remain unchanged and the positive predictive value (86%) might increase, whereas the negative predictive value (100%) might decrease.

There are some limitations to this study. First, we only assessed NAA concentration in a subset of the population, at five of the eight study sites. Nevertheless, this subset used clinical MR scanners from three vendors—Philips, Siemens, and GE Healthcare—thus reflecting the range of systems available in clinical practice. The clinical characteristics, brain injury, and neurodevelopmental outcomes of infants who had thalamic NAA concentration measurements were similar to those who did not.

In this study, measurement of thalamic NAA concentration required a long acquisition duration (approximately 25 min) to accurately measure sources of measurement bias due to pathology (ie, individual metabolite and water T_1_ and T_2_ values). This will not be necessary in future studies, and the acquisition time for NAA concentration can be shortened dramatically (by a factor of at least two), still using clinically available vendor-provided pulse sequences, and without the need for additional research authorisations with any of the three vendors considered (Philips, Siemens, and GE Healthcare). This simple and rapid acquisition protocol has been effectively applied in the HELIX trial (NCT02387385)—which has recruited 408 infants with moderate or severe neonatal encephalopathy from eight different centres in India, Sri Lanka, and Bangladesh and involves clinical scanners from all three vendors—with high-quality proton MRS data obtained throughout.^26^

A second limitation is the large number of participants we excluded prior to DW-MRI analysis. In the absence of a phantom with a well defined ground truth (ie, gold standard) for validating DW-MRI measures in the study, we restricted the analysis to two sites that recruited a large proportion of participants and shared equivalent MR gradient coils and operating software versions. This minimised the likelihood of introducing intersite variance due to acquisition protocol alone. There remains considerable interest in the analysis of DW-MRI data for multicentre multivendor studies in various patient populations, and progress has been made in this area since the initiation of this study.^27^ However, this limitation to our study highlights the difficulty in applying DW-MRI to multicentre studies compared with proton MRS, where more sites can be included.

A third limitation is related to the interpretation of surrogate biomarkers. Although these data show the clear prognostic potential of cerebral MR biomarkers in cooled infants, it is not clear whether a proposed intervention would affect these values, and if so, whether a change in these biomarkers influences an individual's outcome. The only way to test this is through phase 3 interventional trials with the biomarkers alongside the gold standard of careful assessment of neurodevelopmental outcome, and several such multicentre trials are currently in progress.^26^, ^28^

We chose to focus on the thalamus for proton MRS, which could theoretically miss injury specific to other brain regions. The thalamus has exceptionally high metabolic activity in the neonatal brain, and so is disproportionately affected by acute injury.^29^ Furthermore, the clinical impact of thalamic injury is often apparent by 2 years of age. The high prognostic accuracy of NAA concentration in our study, independent of the pattern of visible cerebral injury, supports this notion.

The data from this study have several key implications for research and clinical practice. Typically, neonatal neuroprotection trials are powered for a primary outcome derived from clinical assessments years after the intervention. As a result, such trials require large numbers of participants, and so the smallest early-phase studies will still have a duration of several years.^25^, ^30^ The measurement of NAA concentration could therefore improve the efficiency of neonatal neuroprotective trials in one of two ways. One option is for it to be adopted as a surrogate outcome measure in a phase 2 study to give a rapid go-or-no-go assessment. Such a model was adopted by Azzopardi and colleagues^31^ who did not find any effect of the intervention (Xenon gas) on DW-MRI or lactate–NAA peak-area ratio from MRS. Unlike the latter measure, NAA concentration is normally distributed and more clearly separates between normal and adverse outcomes, while explaining more of the variation in 2-year outcome measures across participants. Furthermore, the MR protocol used in the TOBY Xenon trial^13^ was limited to a small number of 3·0 Tesla scanners from a single vendor (Philips) with closely matched hardware and software specifications, limiting generalisability.

Alternatively, NAA concentration could be used to optimise protocol design before proceeding to a phase 3 trial. For example, several putative adjuvant therapies require careful dose-escalation studies to maximise the likelihood of neuroprotective effects and define an optimal dosing range. One such ongoing multicentre trial (COMET, NCT03409770) is examining thalamic NAA concentration in infants with mild encephalopathy randomly assigned to different cooling durations, so as to model the duration response curve and determine an optimal treatment duration.

Finally, the standardisation and application of harmonised proton MRS protocols across scanners from multiple vendors and across different sites overcomes a key roadblock in the routine clinical use of MRS in neonatal encephalopathy, by providing normal ranges and cutoff values for adverse outcomes. The standardised acquisition and analysis protocols for this study are given in the MR technical summary (appendix). Given that the proton MRS techniques employed in this study are widely available on clinical scanners, they can be added to existing clinical protocols to improve routine prognostication or incorporated into clinical trial protocols to provide surrogate outcome measures.

In conclusion, proton MRS biomarkers acquired soon after birth, particularly thalamic NAA concentration, can accurately predict neurodevelopment 2 years after neonatal encephalopathy. Thus, we believe that all infants with neonatal encephalopathy should receive proton MRS as part of their routine care. Additionally, it can be applied to increase the power of neuroprotection trials while reducing their duration.
