# Supplementary material for: Magnetic resonance spectroscopy assessment of brain injury after moderate hypothermia in neonatal encephalopathy: a prospective multicentre cohort study
Source: Lancet Neurol. 2019 Jan;18(1):35–45. doi: 10.1016/S1474-4422(18)30325-9 (PMC6291458; doi:10.1016/S1474-4422(18)30325-9)
Supplement: Supplementary appendix [file mmc1.pdf]

THE LANCET
Neurology

Supplementary appendix

This appendix formed part of the original submission and has been peer reviewed. We post it as supplied by the authors.

Supplement to: Lally PJ, Montaldo P, Oliveira V, et al. Magnetic resonance spectroscopy assessment of brain injury after moderate hypothermia in neonatal encephalopathy: a prospective multicentre cohort study. *Lancet Neurol* 2018; published online Nov 14. http://dx.doi.org/10.1016/S1474-4422(18)30325-9.

Appendix

Supplementary Table 1: Neurological examination for classifying babies with encephalopathy 2

Supplementary Table 2: MR technical summary (MR spectroscopy and diffusion MRI) 3

Supplementary Table 3: Pattern of injury score on conventional magnetic resonance imaging 7

Supplementary Table 4: Regression coefficients from the final multivariable regression models examining factors associated with the Bayley-III scores and explanation of the multivariate analysis . 8

Supplementary Table 5: Baseline characteristics and brain injury of babies with and without absolute quantification of thalamic N-acetyl aspartate ([NAA]) 9

Supplementary Table 6: Comparison of babies with good and adverse outcome in the subgroup with absolute quantification of thalamic N-acetyl aspartate ([NAA]) 10

Supplementary Table 7: List of collaborators 11

Supplementary Figure 1: Receiver operating characteristic (ROC) curves for selected* prognostic markers... 12

Supplementary Table 1: Neurological examination for classifying babies with encephalopathy

Categories (total 6)	Signs of neonatal encephalopathy (NE) in each category (circle the most appropriate level)			
	NORMAL	MILD NE	MODERATE NE	SEVERE NE
**1. Level of consciousness**				
	Alert, Responsive to external stimuli (state dependent, eg. post feeds)	Hyper-alert, has a stare, jitteriness, high-pitched cry, exaggerated responds to minimal stimuli, inconsolable	Lethargic	Stupor/coma
**2. Spontaneous activity**				
	Changes position when awake	Normal or Decreased	Decreased activity	No activity
**3. Posture**				
	Predominantly flexed when quiet	Mild flexion of distal joints (fingers, wrist usually)	Moderate flexion of distal joint, Complete extension	Decerebrate
**4. Tone**				
	Strong flexor tone in all extremities + strong flexor hip tone	Normal or Slightly increased peripheral tone	Hypotonia (focal or general) or Hypertonia	Flaccid<br><br>Rigid
**5. Primitive reflexes (Circle only the highest level in each sign; The maximum score is only one in any one category)**				
Suck	Strong, easily illicit	Weak, poor	Weak but has a bite	Absent
Moro	Complete	Partial response, Low threshold to illicit	Incomplete	Absent
**6. Autonomic system (Circle only the highest level in each sign; The maximum score is only one in any one category)**				
Pupils	In dark: 2·5-4·5 mm<br>In light: 1·5-2·5 mm	Mydriasis	Constricted	Deviation/ dilated/ non-reactive to light
Heart rate	100-160 bpm	Tachycardia (HR > 160)	Bradycardia (HR < 100)	Variable HR
Respiration	Regular respirations	Hyperventilation (RR > 60/min)	Periodic breathing	Apnoea or requires ventilator
**Total score**				
* Seizure	None	None	Yes / No	Yes / No
Infant who has seizure will be Moderate or Severe NE depending on the neurologic exam. Seizure with normal or mild NE or moderate NE on neurologic exam will be “Moderate NE”. Seizure with severe NE will be “Severe NE”. The level of encephalopathy will be assigned based on which level of signs (moderate or severe) predominates among the 6 categories. If moderate and severe signs are equally distributed, the designation is then based on the highest level in Category #1: The level of consciousness.				

Supplementary Table 2: MR technical summary (MR spectroscopy and diffusion MRI)

MR Spectroscopy

Protocol:

Step	TE (ms)	TR (ms)	Water suppression	Phase cycling	Sub-spectra	Dummy scans	Centre frequency (ppm)
**1**	288	2288	On	8	16	2	2·01
**2**	60	2060	On	8	8	2	2·01
**3**	60	5000	On	8	8	2	2·01
**4**	60	10060	Off	8	1	1	4·67
**5**	124	10124	Off	8	1	1	4·67
**6**	205	10205	Off	8	1	1	4·67
**7**	316	10316	Off	8	1	1	4·67
**8**	495	10495	Off	8	1	1	4·67
**9**	1000	11000	Off	8	1	1	4·67

Representative *in-vivo* spectra:

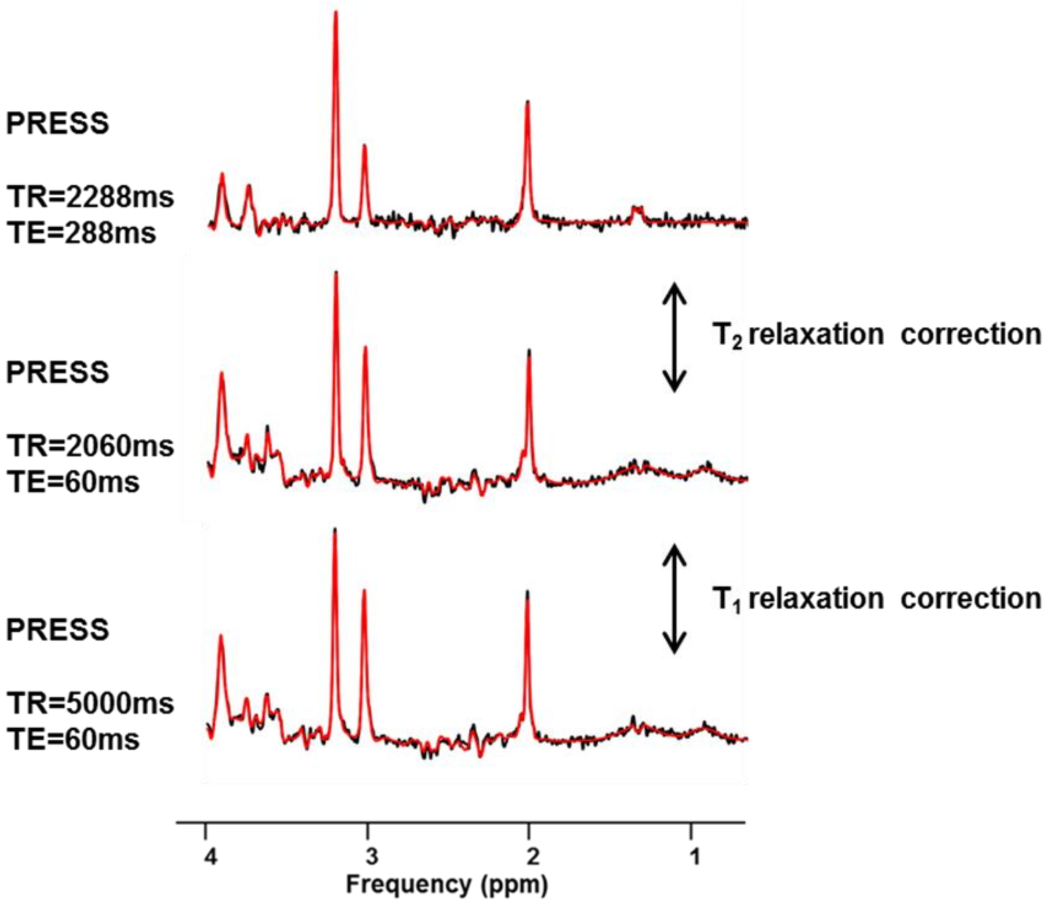

Spectra collected according to the protocol, with the LCModel fit overlaid (in red). These spectra are representative of the mean linewidth across all analysed spectra. The relationship between the spectra at each step (via T_1 or T_2 relaxation effects) is also highlighted.

Quality assurance:

The inter-site variation in MRS measurements was quantified across all scanners using a spherical phantom containing a buffered solution of 10mM N-acetylaspartate (NAA) and 10mM lactate (Lac). *In vivo* quality assurance included the visual assessment of motion from imaging before and after MRS; visual assessment of voxel placement accuracy; assessment of protocol adherence; manual rejection of motion corrupted sub-spectra; automated spectral corrections for both frequency and phase; and the rejection of spectra with linewidths outside the normal distribution of the full dataset.

Analysis:

All water suppressed spectra were analysed using LCModel (v6·3-1J), with basis sets simulated using VeSPA (v0·9·11) with ideal RF pulses according to the PRESS sequence timing employed by each vendor for each acquisition (personal communication). The following metabolites were included in the simulations and analyses: acetate (Act), alanine (Ala), ascorbate (Asc), betaine (Bet), aspartate (Asp), choline (Cho), phosphocholine (PCh), glycerophosphocholine (GPC), creatine (Cr), phosphocreatine (PCr), gamma-aminobutyric acid (GABA), glucose (Glc), glutamate (Glu), glutamine (Gln), glutathione (GSH), glycine (Glyc), lactate (Lac), myo-inositol (mIns), N-acetylaspartate (NAA), N-acetylaspartyl glutamate (NAAG), phosphoethanolamine (PE), propylene glycol (PGC), scyllo-inositol (Scyllo), taurine (Tau), and threonine (Thr). Lipid and macromolecule components were estimated at TE=60ms only, with the default parameterisation in LCModel.

Specific LCModel control parameters were, for TE=288ms:

NSIMUL=0
NCOMBI=17
CHCOMB(17)='Thr+Lac'
PPMST=4·0 (default)
PPMEND=0·2 (default)

And for TE=60ms:

NSIMUL=11
NCOMBI=17
CHCOMB(17)='Thr+Lac'
PPMST=4·0 (default)
PPMEND=0·2 (default)

The methyl peaks of NAA, NAAG, choline (Cho), phosphocreatine (PCr), and creatine (Cr) were separated from other groups in the basis spectra to allow quantification of individual relaxation rates. NAA+NAAG methyl peaks at ~2·0ppm were combined and referred to as 'NAA', and PCr+Cr methyl peaks at ~3·0ppm were combined and referred to as 'Cr' due to strong covariance. Thr+Lac were combined and referred to as 'Lac' due to strong covariance. A single peak was used to fit the choline signal at ~3·2ppm and referred to as 'Cho'. Water unsuppressed signals were quantified using HLSVD. The parenchymal water signal was quantified by biexponential fit of the water unsuppressed series, with a long T_2 component (fixed at 500ms) accounting for mobile water.

NAA/Cho, NAA/Cr and Lac/NAA were all derived from the first acquisition only (TR/TE=2288/288ms).
[NAA] was calculated from the fitted NAA methyl singlets throughout the experiment, comparing the relaxation corrected signal to the relaxation corrected unsuppressed water signal, after partial volume correction. This process is outlined in the following figure:

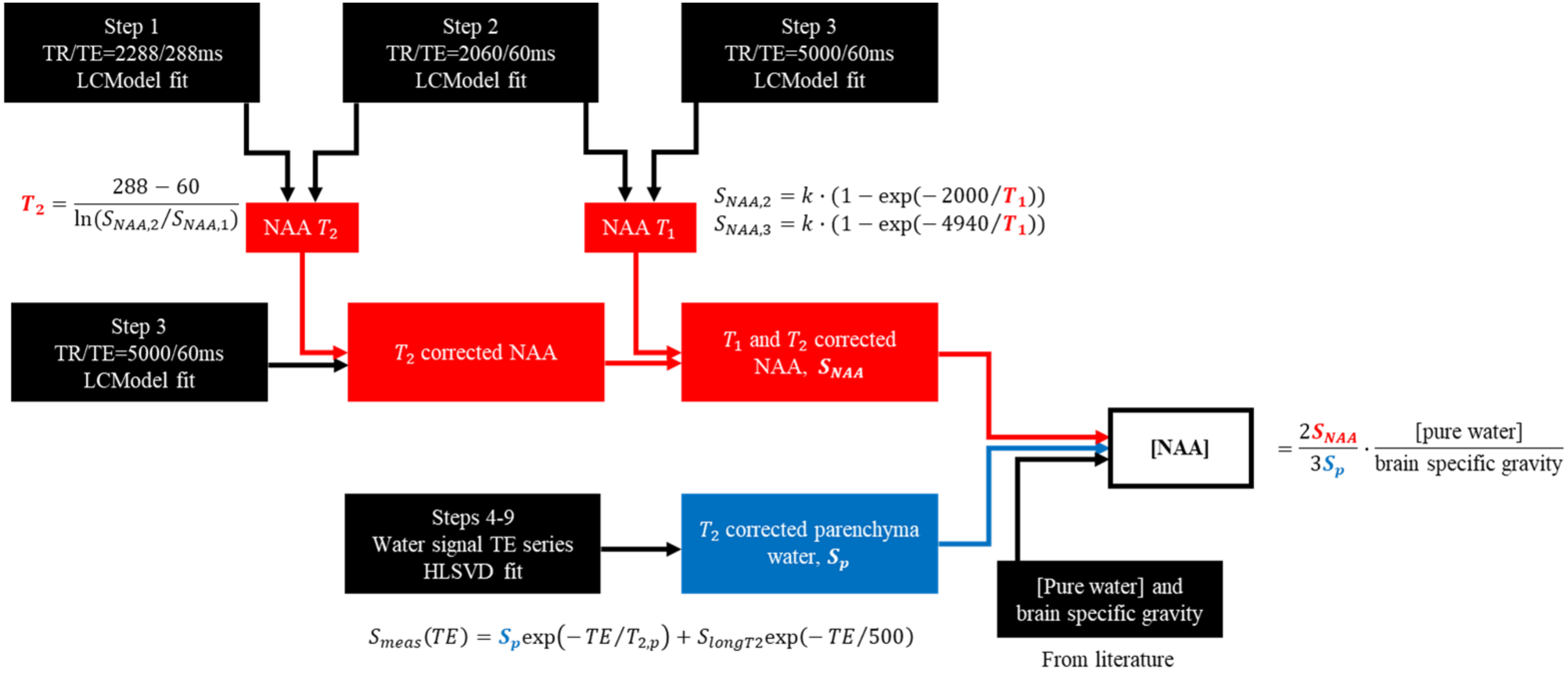

Calculation steps to derive thalamic NAA concentration, [NAA], from the data collected in the various steps of the protocol. Black boxes show the input data, either obtained from the LCModel/HLSVD fits or reference values. Red boxes and text denote calculated quantities from the NAA resonance, and blue boxes and text denote calculated quantities from the water signal. Equations accompany the different calculation steps. $S_{NAA,step}$ denotes the NAA signal intensity in the corresponding protocol step, with the relaxation corrected intensities for NAA and parenchyma water given by S_{NAA} and S_p respectively.

Diffusion MRI

Protocol:
32 direction 2D spin-echo echo-planar imaging, b=0,750 s/mm^2, TE=49 ms, 1·75×1·75×2 mm^3 voxels, parallel imaging acceleration factor 2·0

Quality assurance and processing:
Raw data was assessed for excessive motion and acquisition artefacts, before isolating the brain signal and undertaking corrections for eddy currents and motion using the Functional MRI of the Brain Software Library (FSL v5·0·9, with eddy openmp patch).

Diffusion tensors were then calculated in FSL, and the sum-of-squares error maps inspected for underlying artefacts. Datasets of sufficient quality were spatially normalised to a group-wise template in DTI-TK via rigid, affine and diffeomorphic transformations. Major white matter tracts were selected by thresholding according to fractional anisotropy (FA>0·2) and skeletonised using FSL.

To select a region of interest in the posterior limbs of the internal capsule, the mean FA skeleton was manually segmented to isolate these tracts, as shown below:

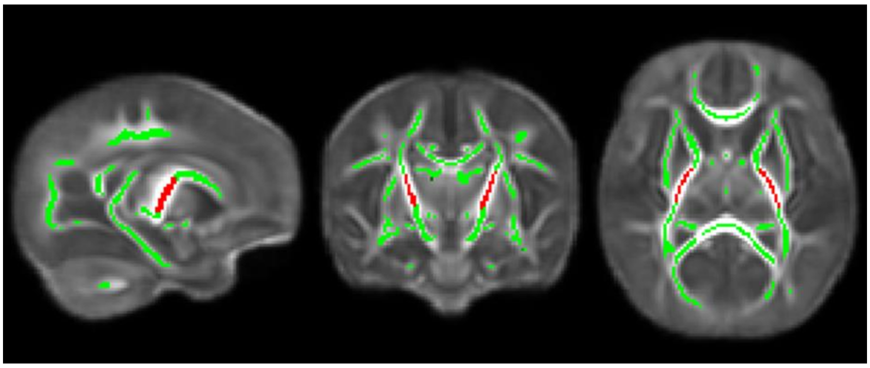

Mean FA skeleton thresholded to include only major white matter tracts (green). Manually segmented PLICs are shown overlaid (red)

The mean FA and standard deviation within this region of interest was then used for assessing prognostic accuracy.

Tract-based spatial statistics (FSL) was then used to examine relationships between FA and neurological outcomes across all of the major white matter tracts included in the mean skeleton. These analyses were corrected for multiple comparisons using threshold-free cluster enhancement.

Supplementary Table 3: Pattern of injury score on conventional magnetic resonance imaging

1. Posterior limb of the internal capsule (PLIC) score: 0=normal, 1=equivocal (reduced or asymmetrical signal intensity), 2=loss (reversed or abnormal signal intensity bilaterally on T_1 and/or T_2 weighted sequences).

2. Basal ganglia and thalamic (BGT) score: 0=normal, 1=mild (focal abnormal signal intensity), 2=moderate (multifocal abnormal signal intensity), 3=severe (widespread abnormal signal intensity)

3. White matter (WM) score: 0=normal, 1=mild (exaggerated long T_1 and long T_2 in periventricular white matter only), 2=moderate (long T_1 and long T_2 extending out to subcortical white matter and /or focal punctate lesions or focal area of infarction), 3=severe widespread abnormalities including overt infarction, haemorrhage, and long T_1 and long T_2. Infarction was classified as areas of excessive long T_1 and long T_2 with either loss of grey-white matter differentiation (usually within first week exaggerated grey-white matter differentiation after the first week). Optic radiation was scored as abnormal if it demonstrated increased signal intensity on T_1 weighted images.

4. Cortical involvement was scored as the presence of abnormal signal intensity, usually decreased T_1 or cortical highlighting. 0=normal, 1=mild (1–2 sites involved), 2=moderate (3 sites involved), 3=severe (more than 3 sites involved). The sites documented included specifically the central sulcus, interhemispheric fissure, and the insula.

Each infant was then given a score for PLIC, BGT, WM, and cortex[13]

Supplementary Table 4: Regression coefficients from the final multivariable regression models examining factors associated with the Bayley-III scores and explanation of the multivariate analysis

Variable	Category / term	Cognitive[a]		Language[a]		Motor[a]	
		**Coefficient (95% CI)**	**p-value**	**Coefficient (95% CI)**	**p-value**	**Coefficient (95% CI)**	**p-value**
**Neurological exam**	None	0	0·05	-		0	0·07
	Mild-Severe	-12 (-24, 0)				-10 (-21, 1)	
**Thalamic [NAA]**	Linear term	17 (3, 30)	0·02	23 (19, 48)	<0·001	18 (16, 41)	<0·001
	Quadratic term	-1 (-2, 0)		-1 (-3, 0)		-1 (-2, 0)	
**PLIC sign**	0	0	0·02	0	0·003	0	0·003
	1	-14 (-25, -2)		-8 (-19, 4)		-10 (-19, 1)	
	2 or 3	-17 (-32, -2)		-24 (-37, -11)		-27 (-40, -14)	
**NAA/Cho**		30 (7, 54)	0.01	-		-	
		R^2 = 57%		R^2 = 59%		R^2 = 68%	

[NAA]: Concentration of N-acetylaspartate; Cho: Choline
PLIC sign: Loss of the normal high signal intensity in the posterior limb of internal capsule on T_1-weighted magnetic resonance imaging

Discharge neurological examination score, Lactate/N-acetyl aspartate, N-acetyl-aspartate concentration, N-acetyl-aspartate/Creatine, N-acetyl-aspartate/Choline, Basal ganglia/thalamic MRI score, white matter MRI score, cortical score MRI score, PLIC MRI score, and amplitude integrated electroencephalography score were included in the regression model.

The multivariable analyses for the Bayley scores adjusted for gestational age and postnatal age. However, these were not adjusted for the analyses involving an adverse outcome. Firstly, our analyses suggested that neither variable was significantly associated with an adverse outcome, and thus is unlikely to confound the relationship between the other variables and this outcome. Additionally, as mentioned in the paper, the strength of association between NAA and an adverse outcome was so strong that almost all variation in the outcome was explained, and it was not mathematically possible to include further predictor variables (such as gestational age) in the model.

For gestational age, the data suggested a mean (±SD) of 39.9 ± 1.5 for those with no adverse outcome, and 39.6 ± 1.4 with an adverse outcome. This was not significant (p=0.27 from unpaired t-test).

For post-natal age, the data suggested a median [IQR] of 7 [5, 10] days for those with no adverse outcome, and 8 [6, 12] with an adverse outcome. This was not significant (p=0.66 from Mann-Whitney test).

Gestational age at birth correlated with NAA (r=0.31, p=0.003), but age at MRI was no related to NAA (r= R=0.02, p=0.83) (Pearson correlation).

Supplementary Table 5: Baseline characteristics and brain injury of babies with and without absolute quantification of thalamic N-acetyl aspartate ([NAA])

	**[NAA] done (n=92)**	**[NAA] not done (n=137)**	**P value*/Mean difference (95% Confidence intervals)**
Birth weight, Kg	3·4 (0·5)	3·4 (0·6)	0·58
Gestation, Weeks	39·8 (1·6)	39·9 (1·4)	0·89
Cord pH	6·9 (2·2)	6·9 (1·7)	0·89
Base excess	-15 (7·6)	-14·3 (5·5)	0·51
Apgar 5 min	4·4 (2·5)	4·2 (2·2)	0·60
Apgar 10 min	5·5 (2·3)	5·7 (2·2)	0·54
Age at the MRI scan, Days	7·8 (3·8)	8·8 (4·5)	0·09
NE stage			
Mild	16 (17)	21 (16)	2·1% (-7·4 to 12·4)
Mod	67 (73)	96 (73)	2·8% (-9·4 to 14·2)
Severe	9 (10)	14 (11)	-0·4% (-8·1 to 8·3)
BGT score			
0	76 (83)	102 (80)	8·2% (-3 to 18·3)
1	6 (7)	14 (11)	
2	8 (9)	9 (7)	
3	2 (2)	6 (7)	
White matter score			
0	30 (33)	47 (36)	-1·7% (-13·7 to 10·8)
1	38 (41)	46 (35)	
2	20 (22)	28 (21)	
3	1 (1)	0	
Cortical score			
0	76 (83)	98 (75)	11·1% (-0·3 to 21·4)
1	12 (13)	21 (16)	
2	3 (3)	4 (3)	
3	1 (1)	8 (6)	
PLIC score			
Normal	76 (83)	106 (81)	5·2% (-5·7 to 15·2)
Equivocal	9 (10)	13 (10)	
Abnormal	7 (8)	12 (9)	
Lactate/NAA†	0·15 (0·09)	0·15 (0·08)	0·8
NAA/Choline	0·82 (0·17)	0·84 (0·14)	0·3; 0·02 (-0·02 to 0·07)
NAA/Creatine	1·5 (0·22)	1·6 (0·29)	<0·001; 0·04 (0·06 to 0·21)
Adverse outcome	12 (15)	19 (18)	-0.8% (-9·5 to 8·8)
Bayley III composite cognitive score	95·9 (20·3)	97·3 (20·4)	0·58; 1·7 (-4·3 to 7·8)
Bayley III composite language score	88·9 (21·1)	90·9 (20·5)	0·54; 3·2 (-4·4 to 8·3)
Bayley III composite motor score	92·2 (20·9)	93·9 (20·5)	0·58; 3·2 (-4·5 to 8·1)

All data are n (%) or mean (SD) unless otherwise specified. *T-test or Mann-Whitney U test
†Median (Interquartile range)
NE: Neonatal encephalopathy; PLIC: Posterior limb of internal capsule; NAA: N-acetylaspartate; Basal ganglia/thalami: 0=normal, 1=mild injury, 2=moderate injury, 3=severe injury; White matter: 0=normal, 1=mild injury, 2=moderate injury, 3=severe injury; Cortex: 0=normal, 1=mild injury, 2=moderate injury, 3=severe injury.

Supplementary Table 6: Comparison of babies with good and adverse outcome in the subgroup with absolute quantification of thalamic N-acetyl aspartate ([NAA])

	Good outcome (n= 78)	Adverse outcome (n=12)	P value*/Mean difference (95% Confidence intervals)
Birth weight, Kg	3·4 (0·5)	3·4 (0·6)	0·48
Gestation, Weeks	39·7 (1·7)	39·4 (1·6)	0·99
Cord pH	6·9 (2)	6·9 (2·9)	0·35
Base excess	-14·3 (6·9)	-18·5 (9·8)	0·26
Apgar 5 min	4·7 (2·4)	2·6 (2·2)	0·02
Apgar 10 min	5·9 (2·3)	4·3 (1·9)	0·07
Age at the MRI scan, Days	7·6 (3·7)	7·7 (3·5)	0·9
NE stage			
Mild	20 (26)	1 (8)	17·3 (-11, 30)
Mod	56 (72)	4 (33)	
Severe	2 (3)	7 (58)	
BGT score			
0	68 (88)	4 (33)	53·8 (24·8, 74·2)
1	5 (7)	2 (17)	
2	4 (5)	4 (33)	
3	0	2 (17)	
White matter score			
0	23 (30)	3 (25)	4·5 (-25·1, 23·9)
1	38 (49)	1 (8)	
2	15 (20)	6 (50)	
3	1 (1)	1 (8)	
Cortical score			
0	64 (83)	8 (67)	15·4 (-6·5, 43·8)
1	12 (16)	2 (17)	
2	1 (1)	1 (8)	
3	0	1 (8)	
PLIC score			
Normal	71 (92)	3 (25)	66 (36·6, 82·8)
Equivocal	5 (7)	3 (25)	
Abnormal	1 (1)	5 (42)	
[NAA], mmol/kg wet weight	7·1 (0·8)	4 (1·3)	<0·001; 3·1 (2·2, 3·9)
Lactate/NAA†	0·14 (0·07)	0·54 (1·1)	<0·001
NAA/Creatine	1·5 (0·17)	1·2 (0·26)	0·003; 0·29 (0·13, 0·47)
NAA/Choline	0·82 (0·16)	0·67 (0·14)	0·004; 0·17 (0·07, 0·26)
Bayley III composite cognitive score	102·5 (12·7)	12 (57·3)	<0·001; 45·2 (39·5, 50·9)
Bayley III composite motor score	99·8 (10·5)	49·8 (10·5)	<0·001; 49·9 (43·0, 56·9)
Bayley III composite language score	96·7 (12·4)	49·8 (8·9)	<0·001; 46·8 (40·5, 53·1)
GMFCS score†	0 (0)	4·5 (1)	<0·001
Cerebral palsy	2 (3)	12 (100)	<0·001
Visual problems	0	4 (33)	<0·001
Hearing problems	1 (1)	2 (17)	0·07

All data are n (%) or mean (SD) unless otherwise specified. *T-test or Mann-Whitney U test
†Median (Interquartile range)
NE: Neonatal encephalopathy; PLIC: Posterior limb of internal capsule; NAA: N-acetylaspartate; Basal ganglia/thalami: 0=normal, 1=mild injury, 2=moderate injury, 3=severe injury; White matter: 0=normal, 1=mild injury, 2=moderate injury, 3=severe injury; Cortex: 0=normal, 1=mild injury, 2=moderate injury, 3=severe injury.

Supplementary Table 7: List of collaborators

First name	Surname	Institution	Country
Peter J	Lally	Imperial College London	UK
Paolo	Montaldo	Imperial College London	UK
Vânia	Oliveira	Imperial College London	UK
Aung	Soe	Medway NHS Foundation Trust	UK
Ravi	Swamy	Imperial College London	UK
Paul	Bassett	Stats Consultancy	UK
Josephine	Mendoza	Imperial College London	UK
Gaurav	Atreja	Imperial Healthcare NHS Trust	UK
Ujwal	Kariholu	Imperial Healthcare NHS Trust	UK
Santosh	Pattnayak	Medway NHS Foundation Trust	UK
Palaniappan	Sashikumar	Medway NHS Foundation Trust	UK
Helen	Harizaj	Medway NHS Foundation Trust	UK
Martin	Mitchell	Medway NHS Foundation Trust	UK
Vijayakumar	Ganesh	Medway NHS Foundation Trust	UK
Sundeeep	Harigopal	Royal Victoria Infirmary, Newcastle	UK
Jennifer	Dixon	Royal Victoria Infirmary, Newcastle	UK
Philip	English	Royal Victoria Infirmary, Newcastle	UK
Paul	Clarke	Norfolk and Norwich University Hospitals NHS Foundation Trust	UK
Priya	Muthukumar	Norfolk and Norwich University Hospitals NHS Foundation Trust	UK
Prakash	Satodia	University Hospitals Coventry and Warwickshire NHS Trust	UK
Sarah	Wayte	University Hospitals Coventry and Warwickshire NHS Trust	UK
Laurence J	Abernethy	Liverpool Women’s NHS Foundation Trust	UK
Kiran	Yajamanyam	Liverpool Women’s NHS Foundation Trust	UK
Alan	Bainbridge	University College London Hospitals NHS Foundation Trust	UK
David	Price	University College London Hospitals NHS Foundation Trust	UK
Angela	Huertas	University College London Hospitals NHS Foundation Trust	UK
David J	Sharp	Imperial College London	UK
Vaneet	Kalra	Wayne State University	USA
Sanjay	Chawla	Wayne State University	USA
Seetha	Shankaran	Wayne State University	USA
Sudhin	Thayyil	Imperial College London	UK

Supplementary Figure 1: Receiver operating characteristic (ROC) curves for selected* prognostic markers.

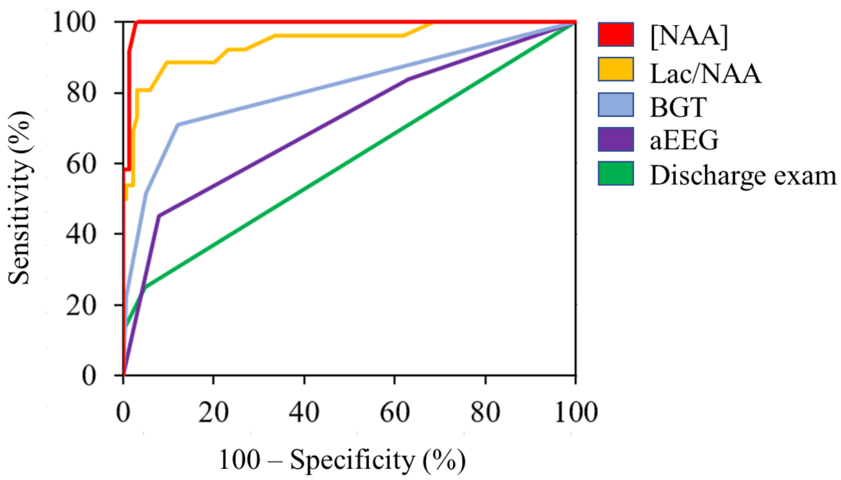

[NAA] = N-acetylaspartate concentration; Lac/NAA = the peak area ratio of lactate and N-acetylaspartate; BGT = basal ganglia/thalamic injury assessed from conventional MRI; aEEG = amplitude integrated electroencephalogram scoring.

*only the ROC curves of the most commonly used biomarkers in a clinical setting are displayed for the sake of clarity. The area under curve of all biomarkers examined is presented in figure 2 in the main text.
